# Effects of ultrasound and ultrasound contrast agent on vascular tissue

**DOI:** 10.1186/1476-7120-10-29

**Published:** 2012-07-17

**Authors:** Steven C Wood, Sible Antony, Ronald P Brown, Jin Chen, Edward A Gordon, Victoria M Hitchins, Qin Zhang, Yunbo Liu, Subha Maruvada, Gerald R Harris

**Affiliations:** 1 Food and Drug Administration, Center for Devices and Radiological Health (CDRH), 10903 New Hampshire Avenue, Silver Spring, MD, 20993, USA; 2 Food and Drug Administration, Center for Drug Evaluation and Research (CDER), 10903 New Hampshire Avenue, Silver Spring, MD, 20993, USA; 3 School of Medicine and Health Sciences, The George Washington University, 2300, Eye Street, NW, Washington, DC, 20037, USA

## Abstract

**Background:**

Ultrasound (US) imaging can be enhanced using gas-filled microbubble contrast agents. Strong echo signals are induced at the tissue-gas interface following microbubble collapse. Applications include assessment of ventricular function and virtual histology.

**Aim:**

While ultrasound and US contrast agents are widely used, their impact on the physiological response of vascular tissue to vasoactive agents has not been investigated in detail.

**Methods and results:**

In the present study, rat dorsal aortas were treated with US via a clinical imaging transducer in the presence or absence of the US contrast agent, Optison. Aortas treated with both US and Optison were unable to contract in response to phenylephrine or to relax in the presence of acetylcholine. Histology of the arteries was unremarkable. When the treated aortas were stained for endothelial markers, a distinct loss of endothelium was observed. Importantly, terminal deoxynucleotidyl transferase mediated dUTP nick-end-labeling (TUNEL) staining of treated aortas demonstrated incipient apoptosis in the endothelium.

**Conclusions:**

Taken together, these *ex vivo* results suggest that the combination of US and Optison may alter arterial integrity and promote vascular injury; however, the *in vivo* interaction of Optison and ultrasound remains an open question.

## Background

Ultrasound (US) is widely used clinically: applications include fetal development monitoring [1] and monitoring of cerebral hemorrhages [2]. The clinical use of US contrast agents produces few side effects and the safety profile drives the technology. US contrast agents have been developed to enhance imaging via the generation of echo signals at the tissue-gas interface and following microbubble collapse. Intravascular ultrasound (IVUS) [3] has revolutionized the imaging of coronary circulation and virtual histology is the product of the IVUS imaging. In addition, trans-esophageal echocardiography studies are frequently used to assess ventricular function. Intravascular gene transfer using microbubbles has been achieved, which has the potential to interdict into disease processes via gene therapy [4].

Optison is a first generation US imaging agent that is comprised of a suspension of microspheres. These microspheres, 3–4.5 uM in diameter (32 uM maximum), consist of the insoluble gas Perflutrene, [5] surrounded by a shell of human serum albumin. US, when coupled with US contrast agents, can have marked effects upon cells and vascular tissue. Notably, hemolysis of red blood cells (RBCs) can occur [6] and tumor ablation by anti-tumor tagged microbubbles and ultrasound has been demonstrated. In addition, microvascular hemorrhaging can be induced by these combination treatments [7] and the blood brain barrier can be disrupted [8].

The clinical use of Optison has resulted in adverse reports. For example, the generation of cardiac arrhythmias is an ongoing concern. Currently, US contrast agents are not recommended in the context of acute coronary syndromes, acute myocardial infarction, or unstable cardiopulmonary disease. Following an US procedure, patients should be electrocardiographically monitored for 30 min [9].

In this study, we investigated the effects of both US and Optison on vascular function in an *ex vivo* in an isolated aorta preparation. In addition, we examined how US and US contrast agents altered vascular morphology and apoptosis. Data from these studies suggest that there may be a potential interaction of US and the US contrast agents on vascular function.

## Methods

### Animals

Sprague Dawley male rats weighing 200–250 g were obtained from Harlan Laboratories, Inc., Indianapolis, IN. Upon receipt, the rats were held for 7 days for acclimation in an AAALAC-approved facility with ad libitum access to food and water. All experiments were performed with the approval of the CDRH IACUC. Rats were housed singly, and the lights were on from 8 AM to 8 PM. For perfusion and removal of the aortas, the rats were anesthetized with isoflurane (Halocarbon, River Edge, New Jersey), the thorax was opened, and the animals were exsanguinated via heart puncture. Next, the heart and lungs were removed en bloc. A ligature was loosely placed at the proximal and distal end of the dorsal aorta and a small incision was made at the proximal end. The distal end was transected beyond the ligature. An 18 gauge intravenous catheter was gently inserted into the incision and care was taken to not introduce the catheter deep into the aorta to avoid damaging the endothelium. The residual blood in the aorta was flushed with cell culture media, RPMI 1640 with 10% FBS (GIBCO, Grand Island, NY), then the aorta was gently perfused with approximately 1 ml of cell culture media either with or without 1% Optison. The lower ligature was tightened during the perfusion, and then the proximal ligature was tightened while the catheter was removed, in an effort to retain media in the vessel. The aorta was then gently removed from the animal and placed in cell culture media for transport to the lab.

Following removal from the animal, the explanted aorta was mounted in an exposure chamber with a 2.5 cm diameter opening bounded by two thin (12 μm) plastic membranes separated by 3 mm. The exposure chamber is described in detail below. The time from tissue harvest to placement in the exposure chamber to sonication was about 20 minutes. The animal protocol was approved by the FDA White Oak Institutional Animal Care and Use Committee.

### US waveform and exposure set up

All ultrasound exposures were performed using a system that simulated a clinical ultrasound beam in pulsed Doppler mode. A spherically focused, 2 MHz transducer having a diameter of 2.5 cm and a focal length of 6 cm (Valpey, Fisher, Hopkinton, MA) was excited by a high voltage pulser-receiver (Gammell Applied Technologies, Exmore, VA) using a four cycle burst and a pulse repetition frequency of 1 kHz. The exposure levels were measured with a spot-poled piezoelectric polymer membrane hydrophone that was constructed in-house [10]. It had an active diameter of 0.5 mm. The hydrophone’s sensitivity calibration was traceable to a national standards laboratory. Ultrasonic pressure–time waveforms were recorded, and temporal peak pressures along with lateral focal beam dimensions were measured. The output was calibrated in terms of the Mechanical Index (MI), a standardized quantity for predicting the potential for mechanical biological effects related to cavitation. The pulse duration and −6 dB focal beam width were 2.3 μs and 3 mm, respectively.

**Figure 1:**
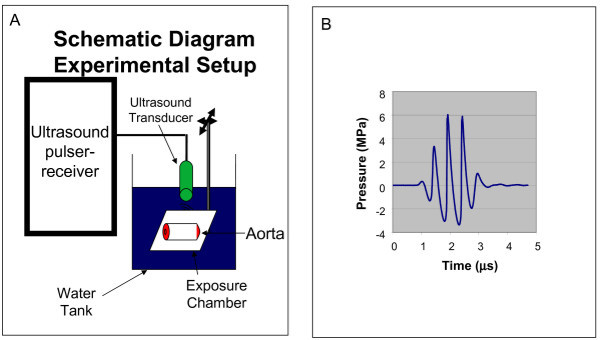
**Ultrasound exposure setup.A**) schematic diagram of the ultrasound transducer, exposure chamber with the dorsal aorta as a target. **B**). the pulse wave used in the experiments.

The exposure tank was 30 cm wide × 60 cm long × 30 cm deep and the water depth was about 20 cm. Sound absorbing rubber was used to minimize reflections. The center of the chamber was positioned at the 6-cm focus of the ultrasound beam via a pulse-echo measurement at low MI (<0.2), and then the exposures were made at MI = 1.9. The exposure system is shown in Figure 1A (transducer and chamber with aorta) along with the temporal pressure waveform in Figure 1B. Aortas were sonified for 30 seconds at 2.5 mm intervals down the length of the vessel.

### US treatment

Aortas were randomly divided into four groups: control, Optison, ultrasound and ultrasound plus Optison. Two aortas were filled with RPMI 1640 and two were filled with 1% Optison in RPMI 1640. After the US or sham exposure, both the control and Optison-treated aortas were removed after five minutes and placed in a separate dish containing cell culture media. One rat dorsal aorta was used per treatment group

### Myobath experiments

The ability of aortas to contract and relax was assessed using a tissue myobath. Briefly, the ligatures to the aortas were cut and the adventitial fat was removed under a dissecting microscope using fine scissors in ice cold oxygenated Krebs buffer. The aortas were cut into 3 mm sections mounted on hooks in 10 ml, water-jacketed myobath vessels (World Precision Instruments, Sarasota, FL). Aortic contraction and relaxation forces were detected using model Fort 25 force transducers (WPI). The force signal was converted into a digital signal via Lab Trax 4/16 (WPI) and recorded via WPI Data Trax 2 2.05 software. Aortas were subjected to a stretch preconditioning by loading them with 0.5 gms for 15 minutes in oxygenated Krebs buffer which was followed by a washout. Krebs buffer was quickly added. Next, phenylephrine (Sigma) dissolved in 0.1 M bisulfite buffer (Sigma) was added to the aortic rings and the bath concentrations ranged from 10^-9^ to 10^-4^ M. Relaxation was induced by the addition of acetylcholine (Sigma) in concentrations ranging from 10^-9^ to 3 × 10^-5^ M. KCL (Sigma), 2 μM final, induced maximal contraction while 10^-5^ M sodium nitroprusside (Sigma) induced maximal relaxation. These experiments were repeated three times and representative results are shown.

### Staining for endothelial markers

Unused 3 mm aortic segments were immediately fixed in 10% formalin/PBS, embedded and sectioned by American Histolabs (Gaithersburg, MD). Sections were dewaxed in xylene (Sigma) and rehydrated in graded ethanol (Pharmaco) solutions for 2 min. Antigen retrieval was performed by microwaving on a high setting for 300 seconds in 0.1 M citrate buffer (Sigma). The slides were blocked with 5% horse sera for 20 min at room temperature, which was provided in the Impress Kit (Vector labs, Burlingame, CA). Following a washing with TBST, anti-VEGF, anti-FLT-1 and anti-FLK-1 (Santa Cruz Biotechnology, Santa Cruz, CA) were diluted 1:50 in 0.1% BSA/HBSS, and placed on the aortic sections. A coverslip was added and the slides were incubated overnight at 4°C. Next, the slides were washed three times with TBST and the slides were treated with 0.03% H_2_O_2_ in ethanol for 15 min. The slides were washed three times with TBST and strepavidin HRP (Jackson Immunoresearch, West Grove, PA), diluted 1:500 in TBST, was pipetted onto the sections. Following a one hour incubation, the slides were washed and diaminobenzidine (DAB) (Vector Labs) was prepared. Following a10 minute incubation, the slides were quenched with distilled water.

### Apoptosis

TUNEL assay was performed according to the manufacturers’ directions (Genscript Corporation, Piscataway, NJ). The slides were de-waxed with xylene and dehydrated with ethyl alcohol. The slides were treated with Proteinase K and then the endogenous peroxidase was inhibited by 3% peroxide (Sigma) in methanol (Sigma). The TUNEL reaction mixture was prepared and added to the slides for 1 hour. Following washing, streptavidin HRP was added for 45 minutes at 37°C. After washing the slides, DAB substrate (Vector Labs) was added with nickel enhancement for ten minutes. Finally, the slides were immersed in water and counter-stained with Hemotoxylin. Images were captured as described above.

### Statistics

All values are expressed as means ± SE. Differences between individual mean values were determined using ANOVA and Dunnett’s test using SAS for Windows (SAS, Cary, NC). Values of p less than 0.05 were regarded as significant.

## Results

**Figure 2:**
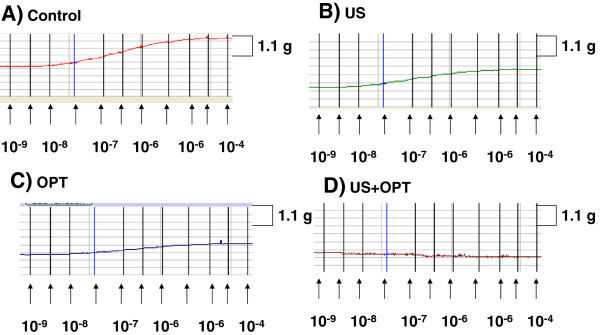
**Isometric force recordings of phenylephrine-induced contraction: A) control, B) US, C) OPT and D) US + OPT.** A representative tracing of three experiments is shown.

**Figure 3:**
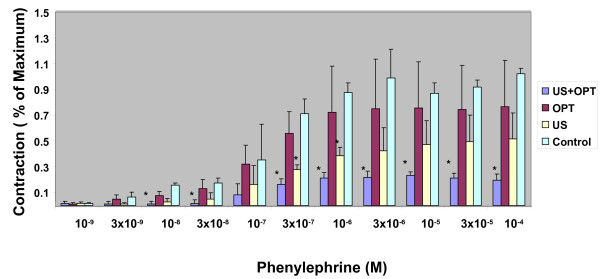
**Effect of US and US contrast agent upon phenylephrine-induced aortic contraction.** Results are expressed as a percentage of the phenylephrine contraction in the control aortas. N = 3; Significant differences of treated vs. control * = p < .05. The results are representative of three experiments.

Vascular contraction and relaxation studies were used to assess the effects of US and US contrast agent on arterial function. The contractile response to phenylephrine was examined initially. As shown in Figure 2, contraction of a representative segment of the aorta was compromised by either US or the US contrast agent. The combination of US and US contrast agent dramatically altered the contractile response. When the response was normalized to % of control (Figure 3), a marked reduction in the contractile response was noted in a statistically significant manner, implying that smooth muscle function was impaired.

**Figure 4:**
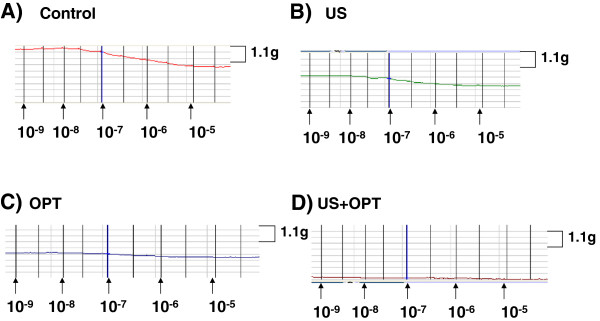
**Isometric force recordings of acetylcholine-induced relaxation: A) control, B) US, C) OPT and D) US + OPT.** A representative tracing of three experiments is shown.

**Figure 5:**
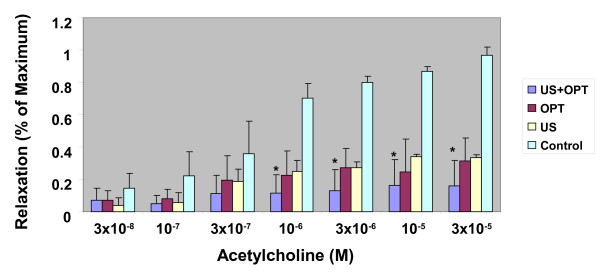
**Effect of US and a US contrast agent upon acetylcholine-induced aortic relaxation.** Sodium nitroprusside was used to induce maximal relaxation. N = 3; Significant differences of treated vs. control * = p < .05. The results are representative of three experimentssections from two experiments are shown.

The ability of aortic segments to relax was examined next. The rings were exposed to increasing concentrations of acetylcholine. In the representative results shown in Figure 4, the control aortas readily relaxed, while the degree of relaxation was reduced in both US- and contrast agent-treated aortas. The combination of US and contrast agent had a marked effect upon relaxation. When the data were normalized to % of control, the reduced vascular response to acetylcholine was apparent and was statistically significant in the US + contrast agent group. Given that relaxation, as shown in Figure 5, is dependent upon the release of soluble factors from endothelial cells, it can be inferred from these data that the endothelium was damaged following the combination treatment.

**Figure 6:**
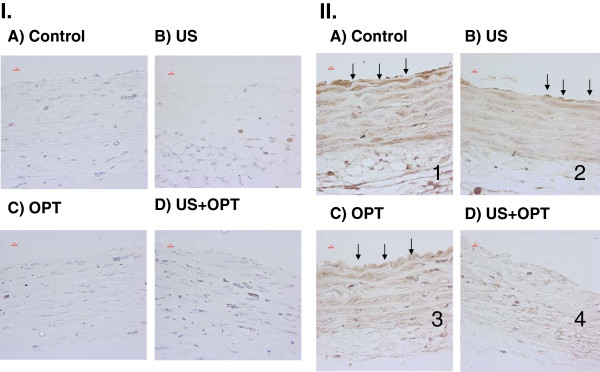
**Assessment of endothelial integrity by immunohistochemistry.** Aortic sections were stained with three endothelial markers, anti-VEGF, anti- FLT-1 and anti-FLK-1 and the epitopes were revealed by DAB staining **I**) Negative control, no primary antibodies, **II**) Treated sections: **A**) control, **B**) US, **C**) OPT and **D**) US + OPT; The short arrows mark the endothelium. Magnification is 40x and representative sections from two experiments are shown.

To assess endothelial integrity, the sections from control and treated aortas were stained with a combination of the FLT-1, FLK1-1 and anti-VEGF. FLT-1 and FLK1-1 are receptors for VEGF, and VEGF is synthesized and released by endothelial cells. Prominent DAB endothelial staining was observed in control sections. Aortas treated with both the US and US contrast agents showed a reduction in staining. Again the combination of US and US contrast agent suppressed the expression of endothelial markers, which is shown in Figure 6.

**Figure 7:**
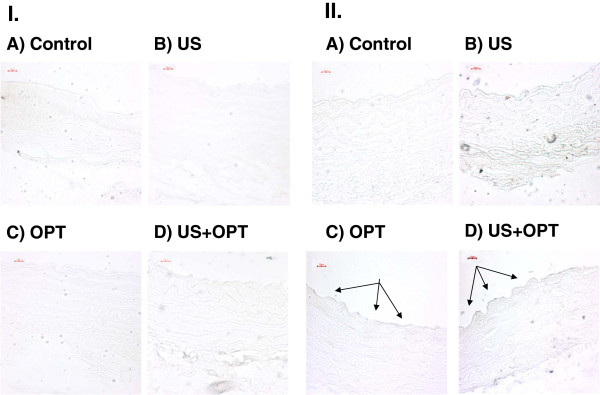
**TUNEL assay of arteries following US and Optison treatment. Aortic sections labeled with UTP-biotin were stained with strepavidin DAB: I) No UTP-biotin II) UTP-biotin is present: A) control, B) US, C) OPT and D) US + OPT; Arrows point to regions under going apoptosis.** Magnification is 40x and representative sections from two experiments TUNEL assay of arteries following US and Optison treatment. Aortic sections labeled with UTP-biotin were stained with strepavidin DAB: **I**) No UTP-biotin **II**) UTP-biotin is present: **A**) control, **B**) US, **C**) OPT and **D**) US + OPT; Arrows point to regions under going apoptosis. Magnification is 40x and representative sections from two experiments are shown

Apoptosis may be responsible for the alteration of the endothelial layer. Sections were subjected to TUNEL analysis. No TUNEL staining was seen in the control and minimal endothelial staining was seen in the US and US + contrast agent-treated aortas. In contrast, TUNEL staining of the endothelial cells was seen when US is combined with US contrast treated aortas, as shown in Figure 7.

## Discussion

Ultrasound contrast agents are widely used for diagnostic ultrasound imaging procedures, but questions have been raised about their safety based on the results of *in vitro* and animal studies. Notably, the combination of US and US contrast agents has been shown to produce vascular damage. A number of these studies report damage to endothelial layer of blood vessels exposed to US and US contrast agents. In this paper, we have explored the physiological consequences of endothelial damage following combined exposure to US and Optison. When combined, this treatment can have marked vascular effects in an *ex vivo* vascular preparation.

As shown in Figures 2 and 3, a marked reduction in contractile response was noted when Optison was combined with US treatment. Phenylephrine is an-adrenergic agonist that induces smooth muscle contraction. The inability of the aortic segment to contract implies that there is a profound injury to smooth muscle. This observation is in agreement with an earlier study which showed dose-dependent smooth muscle damage in *ex vivo* porcine carotid arteries exposed to both US and Optison [11]. Similarly, cardiomyocyte viability has also been compromised by the combination of US and Optison following *in vivo* exposure [12].

Relaxation of arteries by acetylcholine is due to the release of soluble factors from endothelium. As shown in Figures 4 and 5, impaired relaxation was noted when US and Optison were combined. Brayman et al. noted that endothelial monolayers were readily damaged by US and Optison [13]. In rabbit ears, adherence of platelets was observed following US and Optison treatment. Platelet aggregation and injury to endothelial cells were more severe when the contrast agent and US were combined [14]. Importantly, US and Optison can induce marked microvascular damage in rat mesentery [7,15].

Endothelial cells synthesize and release multiple forms of VEGF, which is required to maintain vascular integrity as well to promote angiogenesis [16]. The most common forms of VEGF interact with tyrosine coupled receptors, FLT-1 and FLK-1, on the surface of the endothelium to modulate function in an auto-regulatory fashion. Staining for these three entities was markedly reduced in the US plus Optison group (Figure 6). A slightly decreased DAB signal was seen in either US or Optison treatment.

In this study, endothelial apoptosis was noted during the combined treatment of US and Optison (Figure 7). Importantly, endothelial apoptosis has been found in rabbit corneal endothelial cells treated with both US and Optison *in vivo*[17]. Using confocal microscopy, US and Optison was observed to induce apoptosis at low energy. As emphasized in their study, damage was limited to the endothelial layers, and the internal elastic lamina may protect the smooth muscles cells from inertial cavitation [11]. It should be noted that agents such as Optison have the propensity to bind to activated endothelial cells. If the binding of Optison takes place *in vivo* as well, atherosclerotic lesions may be at risk during US imaging.

Optison, in conjunction with US, has been used for gene therapy, drug delivery, angiogenesis studies, imaging vascular injury and evaluating cardiac function. As with any therapy, there are often untoward effects that need to be balanced the potential benefits. Given that there is the potential for widespread collateral effects, preclinical evaluation of ultrasound contrast agents is warranted which is consistent with the results presented in Figures 3, 5, 6 and 7.

The goal of US directed gene therapy is to identify and transfect selected anatomical structures with the gene(s) of interest. Vascular beds are ideal targets given the relative ease in identification and the delivery of the DNA of interest to the relevant tissues. The transfer of genetic material such as plasmid into a cell requires the brief disruption of the membrane. Additionally, signaling pathways such as ERK are activated and may play a role in the eventual expression of the transfected DNA, probably through mechanical sensing via integrins. US activates ERK ½ signaling via rock in skin fibroblasts [18].

Both endothelial cells and smooth muscle cells can be transfected with plasmids by Optison and US [19]. C-myc expression was decreased following transfection with anti-sense morpholino oligomers in porcine arteries treated *ex vivo*[20]. Neointimal proliferation was inhibited following balloon injury when anti-sense p53 plasmids or decoy E2F decoy oligo nucleotides, Optison and ultrasound were used to transfect rat carotid arteries [21,22]. The contractile response to prostaglandin was reduced in porcine carotid arteries that were transfected with eNOS *ex vivo*[23]. Plasmid DNA and viruses can be transduced into skeletal muscle through an intra vascular route using US and US contrast agents [24]. In sum, the use of both Optison and US enhances the nonviral gene transfer is an alternative to using viral vectors [25]. While these studies focused upon transfer of genetic material, there was no attempt to evaluate untoward thrombus formation.

Inflamed endothelial cells express cell adhesion molecules such as Intracellular adhesion molecule (ICAM) and vascular cell adhesion molecule (VCAM). Both ICAM and VCAM can be used to image inflammation and the inflamed tissue can be targeted for *in vivo* drug delivery as well. A gas-filled microbubble with anti-ICAM-1 antibody on its shell specifically binds to activated endothelial cells over expressing ICAM-1 [26]. Significantly, the endothelial BBB can be altered by ultrasound and contrast agents and may be a means of delivering drugs to the CNS due to altered permeability [27,28]. Other vascular effects include micro vessel rupture, and cell death in the rat spinotrapezius muscle using Optison [29]. Further, petechiae, and capillary leakage was observed in the mouse abdomen following use of Optison [30]. Clearly, the coupling of integrin specific MAB to US contrast reagents may generate tissue specific contrast reagents but, there are nonspecific and collateral damage that results from the combined use of Optison and Ultrasound.

Tumors are dependent upon the formation of neo-vessels for continued growth. Noninvasive, *in vivo*, imaging of vasculature is extremely important for identifying tumors. Most importantly, this permits a means of screening anti-tumor regimens in preclinical models and clinical applications. These imaging techniques also permit high-resolution, volumetric assessments of tumor vascularity. In a preclinical model of breast cancer is shown that correlates with other ultrasonographic measures of blood flow, which may provide greater sensitivity to the microvasculature in real time [31]. The endothelium of tumor neo-vessels express vascular cell endothelial growth factor receptor 2 (VEGFR2). UCA MicroMarker has been with conjugated to anti VEGFR2 have been used to follow angiogenesis in a preclinical murine model for breast tumors [32]. In a human xenograft melanoma model, immuohistochemical COX-2 staining of excised tumors correlated with the contrast-enhanced ultrasound image [33]. The imaging of angiogenesis is dependent upon the expression of tumor or endothelial markers such as VEGFR2. The expression of VEGFR2 may be variable, depending upon the growth stage/size and in humans, clonality. These imaging techniques also permit high-resolution, volumetric assessments of tumor vascularity. The utility of following VEGF receptor and signaling kinases as a marker of endothelial integrity is amply demonstrated in this paper.

Inappropriate thrombus formation in the heart, brain or in a peripheral site is the hallmark of vascular disease. Imaging thrombus is an important application of Optison and US. Abciximab, which recognizes glycoprotein IIb/IIIa receptor was conjugated to Optison and the immuno bubbles enhance the image of arterial thrombus *in vivo *[34]. In contrast, US and USCA can be used in combination to break apart moderate sized clots [35]. In the rabbit ear, US and contrast agent were directed against the auricular vein. When fibrinogen was administered, the vein was occluded by an a thrombus [36]. These examples illustrate the usefulness in identifying thrombus as well as inducing thrombus to inhibit blood flow to a target lesion. The loss of vascular function that we observed in this study illustrates the collateral damage that can be induced.

IVUS imaging of coronary and peripheral arteries is extremely useful technique to image plaque formation and vessel patency. Vulnerable plaque is an arterial lesion that has a propensity for rupture and thrombus formation. IVUS and contrast agent permits the visualization of vasa vasorum density and a combination of lipid core, cap thickness and calcification may help identify the plaques most likely to rupture [37].

Valvular stenosis can easily be visualized by ultrasound examination of the heart. The micro bubbles enhance the image of the ventricle making it easier to identify thrombus, calculate the volume of ejected blood and visualize wall motion. These functional studies are crucial for the clinical assessments of patients [38].

There are contradictory reports regarding the effect Optison on human cardiac function [39]. Perventricular contractions (PVCs) were noted in the human heart [40]. Troponin T was elevation was seen with Optison however, there were no negative histologic findings were seen [41]. In an other study, no changes were seen in PVCs, Troponin I, CK, CK-MB in the human heart [42].

In a preclinical model using the rat heart, PVCs, and microvascular leakage was noted with Optison [43,44]. Importantly, micro lesions were seen histologically with inflammatory infiltrates 24 hours post exposure in the rat heart [44,45]. In glass catfish model, US and USCA revealed focal damage in the tail of fish. Importantly, this was a real time assessment of the damage, which was significant [46]. Our results from this study are consistent with vascular damage that may contribute to the arrhythmias seen *in vivo*.

Rat hearts were subjected to both US and Optison, and cardiac arrhythmias were induced. Cessation of US treatment reversed the effect. However, no histological effect was seen [47]. Rat hearts were treated with ultrasound and Optison, and the RNA was prepared for microarray analysis. The only gene up-regulated was carbonic anhydrase, so there was not dominant gene induction [48].

Use of ultrasound contrast agents, newly-developed microbubble-based products which are administered intravenously to enhance the ultrasound image quality, present new challenges with regard to clinical safety because of the locally destructive forces of inertial cavitation caused by the interaction of ultrasound with the micro bubbles. These destructive forces can damage the endothelium and smooth muscle of the vascular wall. The target patient population that may be exposed to microbubble/ultrasound is large and continues to grow as new applications and new products in this class are developed.

For example, increased permeability due to contrast agent-induced vascular damage can capitalized upon therapeutically to deliver genes and other large molecules across endothelium. However, an adverse event that may occur from microbubble-induced vascular lesions may be the initiation or acceleration of atherosclerotic progression. Since this modality is being considered for delivery of drugs and genes, an even larger patient population (who originally had no cardiovascular disease) will be exposed to long-term risks. Therefore, it is critical to identify the ultrasound and microbubble exposure conditions which cause damage to the vascular endothelium and determine whether microbubble-induced vascular damage increases the risk of atherosclerosis in selected populations.

There are several limitations in the present study: First, it was performed *ex vivo* under static conditions with no blood flow. Second, only one concentration of Optison was employed. In earlier studies (Miller, Dou, and Song 601–07; [6,12,39,49], the microbubble concentration ranged from 0.01% to 2% and maximal RBC hemolysis was seen at 1%. In our experiment, we used 1% Optison, so we are using concentrations that are consistent with previous work. In clinical use, the recommended doses range from 0.5 to 5 ml of Optison infused in a 10 min period.

For these studies, we used a 2 MHz US wave employing a four-cycle tone burst simulating a pulsed Doppler mode and having an MI of 1.9, the maximum setting available on a clinical imaging unit. It is not clear whether similar effects could occur at MI values less than 1.9.

In conclusion, 2 MHz US with an MI of 1.9 and 1% Optison altered contraction and relaxation in rat dorsal aortas exposed *ex vivo*. The changes in arterial function may be due to damage of the endothelium and smooth muscle. This study provides insight into functional parameters of vascular function that may be compromised by US and Optison treatment.

## Competing interests

The authors declare that they have no competing interest.

## Authors' contributions

SCW designed the experiments, performed statistical analyses, and drafted the MS. SA performed the myobath and immunohistochemical procedures. RB assisted with experimental design and US exposures. JC helped conceive the project and revised the MS. EAG constructed the US exposure device, US calibrations, US exposure. YL and SM assisted with the US exposures. GRH designed and calibrated the US exposure device. GRH also assisted with the US exposures. VMH and QZ assisted with the experimental design. All authors read and approved the manuscript.
